# RDA and REA Values of Commercially Available Toothpastes Utilising Diamond Powder and Traditional Abrasives

**DOI:** 10.3290/j.ohpd.a45085

**Published:** 2020-09-04

**Authors:** Blend Hamza, Thomas Attin, Claudia Cucuzza, Andrea Gubler, Florian J. Wegehaupt

**Affiliations:** a Resident, Center of Dental Medicine, University of Zurich, Clinic of Conservative and Preventive Dentistry, Zürich, Switzerland. Performed the experiment, wrote the manuscript.; b Professor and Director, Center of Dental Medicine, University of Zurich, Clinic of Conservative and Preventive Dentistry, Zürich, Switzerland. Critically evaluated the experiment and the manuscript.; c Laboratory Technician, Center of Dental Medicine, University of Zurich, Clinic of Conservative and Preventive Dentistry, Zürich, Switzerland. Performed the experiment.; d Head, Department of Preventive Dentistry and Oral Epidemiology, Center of Dental Medicine, University of Zurich, Clinic of Conservative And Preventive Dentistry, Zürich, Switzerland. Conceived and designed the experiment, critically evaluated the manuscript.

**Keywords:** abrasivity, diamond powder, RDA, REA, toothpastes

## Abstract

**Purpose:**

To investigate whether toothpastes with diamond powder vs those with traditional abrasives abrade dentin and enamel differently and to determine the relative dentin abrasivity (RDA) and relative enamel abrasivity (REA) values of those toothpastes.

**Materials and Methods:**

Dentin and enamel samples of bovine permanent incisors were randomly allocated into groups of eight, brushed with 20 different toothpastes (three of which contained diamond powder) and analysed for their RDA and REA values.

**Results:**

Toothpastes with diamond powder exhibit low RDA values but high REA values. Some RDA values exceeded the ones declared by the manufacturer.

**Conclusion:**

Diamond powder as an abrasive might have a mild action on dentin, but it is highly abrasive on enamel.

Obtaining clean, white teeth is one of the major reasons why people practice daily oral hygiene. In order to meet this demand, manufacturers have saturated the market with a large variety of toothpastes which promise consumers just that. It is well known that mechanical abrasives of different kinds, shapes, sizes and amounts give toothpastes their cleaning property. However, they are also known to cause a certain amount of tooth wear, a property referred to as abrasivity.^1,5,7^ Since abrasives possess both beneficial and hazardous effects, toothpaste abrasivity has been monitored for decades to control these effects.

Established in the 1950s, relative dentin abrasivity (RDA) was the first standardised parameter to determine abrasive potential of toothpastes. It is widely known and accepted as the gold standard.^9,14^ To determine RDA, sound radioactive dentin is brushed with the tested toothpaste. The resulting release of radioactive dentin is then measured and compared to that caused by brushing the same radioactive dentin with an abrasive standard. The abrasivity of the standard abrasive is arbitrarily given the value 100. The abrasivity of the tested toothpaste is then expressed as a percentage of the above-mentioned value. In other words, a toothpaste which causes half the abrasion as the standard abrasive would have an RDA value of 50, i.e. the higher the RDA value, the higher the abrasivity.^8,14^

Relative enamel abrasivity (REA) describes the abrasive potential of a toothpaste on dental enamel. Studies have shown that toothpaste RDA values could not predict their REA values.^4,20^ To determine the REA of a toothpaste, the same method and the same standard abrasive is used as in RDA. Nevertheless, the standard abrasive in REA measurement is arbitrarily given the value 10 (compared to 100 in RDA). The majority of toothpastes on the market are not tested for their REA and only show an RDA. This can be attributed to the fact that dentin is softer than enamel, resulting in a significantly faster substance loss of dentin than enamel. Consequently, especially the abrasion of dentin is a problem clinically. Furthermore, the disregard of REA could also be attributed to the fact that abrasives used in early toothpastes exhibited much lower hardness than enamel and were therefore not assumed to be able to abrade it.^10^ However, some recently marketed toothpastes utilise diamond powder as abrasives, which is much harder than enamel.^18^

Under healthy clinical conditions, the enamel is the first substrate to be contacted by toothpastes. Seen from a primary preventive perspective, REA should also be taken into consideration.

The considerable increase of both incidence and prevalence of tooth wear and the indisputable influence on it of toothpaste abrasivity reveals the need for thorough understanding of the mechanical properties of commercial toothpastes. This is of utmost importance for dentists seeking to provide their patients with sound advice regarding their oral hygiene practice.^11^ It is also important that the properties and qualities of toothpastes and other oral hygiene products be regularly monitored by independent parties. The most recent study concerning this matter in Switzerland was published in 2015, where the RDA of 15 market-leading toothpastes was determined.^17^ However, REA was not evaluated in that study.

The aim of the present study was to determine the REA and RDA of 20 toothpastes and investigate whether toothpastes utilising new abrasive particles, namely diamond powder, behave differently on dentin and enamel.

## Materials and Methods

### Specimen Preparation and Toothpastes

**Fig 1:**
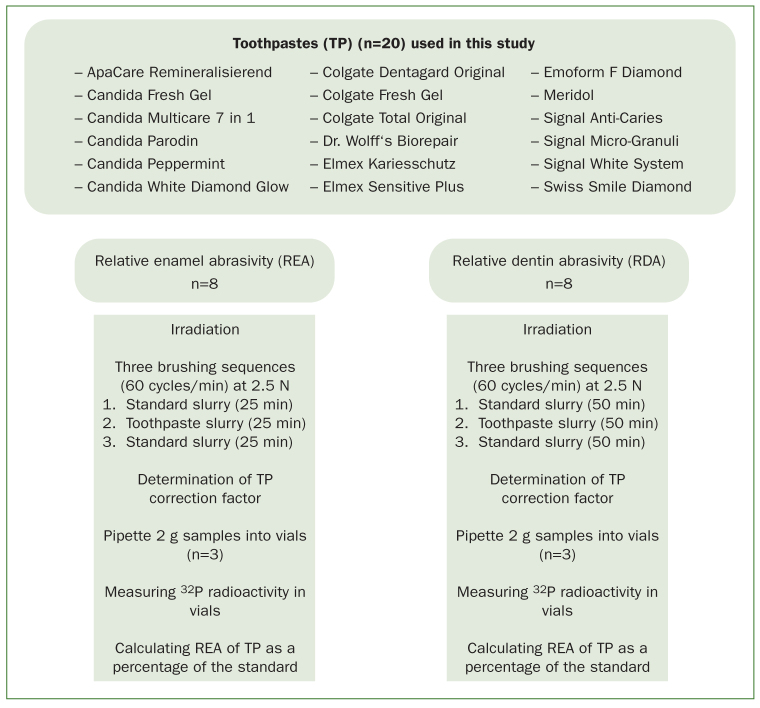
Study design.

**Fig 2:**
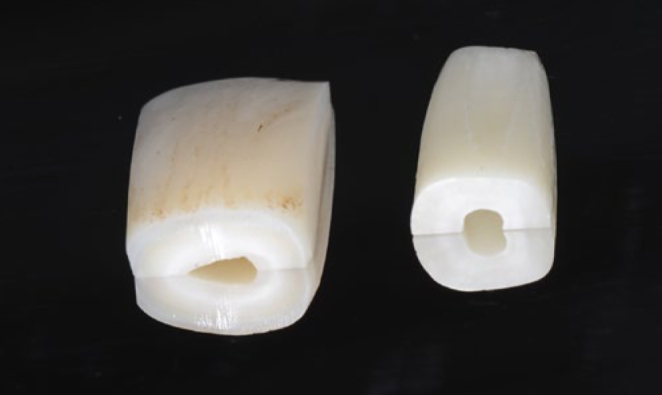
Enamel (left) and dentin (right) specimens.

Twenty toothpastes were purchased from supermarkets, pharmacies or online providers in Switzerland between December 2018 and January 2019 (Fig 1). For each brand of toothpaste purchased, all tubes had the same lot number. Permanent incisors were extracted in the laboratory from bovine mandibles obtained from a slaughterhouse. The incisors were cleaned using scalpels and dental scalers. Roots and crowns were separated using a diamond disk under constant water cooling. They were then sliced through their longitudinal axis (approximately through their root canals), creating a planar surface to facilitate positioning in the embedding material. Dentin surfaces were then polished under water cooling using light blue and light yellow Sof-Lex Pop-On disks (3M Oral Care; St Paul, MN, USA). Polishing was carried out for 2 min using a pressure gauge to maintain the load at 40 to 60 g. Enamel surfaces were first polished for 1 min with a blue Sof-Lex disk, after which they underwent the same polishing procedure as dentin (Fig 2). New disks were used to polish each sample. The pre-trimmed and polished samples from bovine teeth (roots for RDA and crowns for REA) were sent to the Atomic Institute, Vienna, Austria, along with synthetic apatite (Himed; Old Bethpage, NY, USA) for standardisation to be bombarded with neutrons at a maximum temperature of 55°C. This irradiation converts part of phosphorus 31 in hydroxyapatite to radioactive phosphorus 32 (^32^P), which later served as the index of abrasion. After neutron bombardment, specimens were shipped to a type B laboratory (mid-level radiation precautions) in Zurich. To fit in the V-8 cross-brushing machine (Sabri Enterprises; Downers Grove, IL, USA), all specimens were embedded in acrylic resin (Paladur, Heraeus Kulzer; Hanau, Germany) using a Teflon mold and a positioning guide. This ensured the positioning of the specimen surface 1 mm above the surface of the acrylic resin. Care was always taken to prevent any specimen from dehydration. After polymerisation, any remaining overhanging acrylic resin was manually removed with a clipper to fit exactly in the V8 cross-brushing machine.

A total of 10 groups were formed. Each group consisted of 8 randomly selected and numbered specimens. Specimens of all groups were then stored in distilled water until further use.

### Brushing Sequences

To measure the REA and RDA of each toothpaste, each group of specimens (n=8) was subjected to three sequences of brushing, using the ‘sandwich technique’ described by Imfeld et al.^15^ The sandwich technique allows the usage of two toothpastes in one group. There was no control group per se, but a control brushing sequence. The sandwich technique includes two control brushing sequences for each toothpaste.

The samples were first brushed with the standard slurry (RDA 100, REA 10), then with the tested toothpaste slurry, and subsequently again with the standard slurry. The standard slurry contained silica abrasive particles (Sident, Evonik Degussa; Essen, Germany), carboxymethylcellulose, glycerol, a silicon antifoaming agent, and a saliva substitute of a similar buffering capacity as human saliva. The slurry of the tested toothpaste was prepared by mixing 225 g of it with 360 g of the above-mentioned saliva substitute along with 0.45 g of silicon antifoaming agent. All slurries were freshly prepared and well mixed (5 min) before each sequence.

A brushing sequence began by mixing the slurry and positioning dentin (RDA) or enamel (REA) specimens in the 8-place cross-brushing machine (V-8 Cross Brushing Machine). Standard toothbrushes (Paro M43, Esro; Thalwil, Switzerland) were then fixed in the machine. Using a spring gauge, the force applied by the brushes on the specimens was set at 2.5 N. The slurry – whether standard or toothpaste – was poured into 8 tubes in equal amounts. The filled tubes were then stretched tight in the brushing machine with the attached specimens and the brushing sequence was started. All specimens were constantly covered with the slurry throughout the brushing sequence. Abrasive particles were prevented from settling on the bottom of the tube by fitting a silicon paddle to the reciprocating arm of the brushing machine, which ensures constant mixing of the slurries. Each brushing sequence consisted of 30-mm excursions at rate of 60 cycles/min. One cycle refers to a complete forward and backward movement of the brush over the specimen. For RDA measurement, each brushing sequence lasted 25 min (total 1500 cycles). For REA measurement, each brushing sequence lasted 50 min (total 3000 cycles). Between brushing sequences, the brushing machine was thoroughly cleaned using distilled water. New tubes and new brushes were used for each brushing sequence.

### RDA and REA Measurement

^32^P radioactivity was measured in a Tri-Carb A 2700 liquid scintillation analyser (Perkin Elmer; Waltham, MA, USA). At the end of each brushing sequence, the radioactive slurry in each tube was mixed for 1 min to ensure homogeneous distribution in the tube. Three 2-g samples were taken from each tube and pipetted into three glass vials to measure ^32^P radioactivity. In other words, 24 vials (three vials for each of the 8 tubes) were measured for each brushing sequence. In addition to the slurry, each vial received 1 ml of 2M HCl and 12 ml of distilled water. The liquids inside the vials were allowed to rest for at least 24 h least before measuring ^32^P radioactivity. Each vial was measured in the analyser for 60 min or until a standard deviation (σ) of 2% was reached. The analyser measures ^32^P radioactivity in counts per minute and converts them to decays per minute. To compensate colour quenching, the correction factor for each toothpaste slurry as well as the standard slurry was determined and included in the results. Data collected from the two brushing sequences with the standard slurry per specimen were averaged and normalised to the value 100 for RDA or 10 for REA. RDA and REA of the tested toothpastes were then expressed as a percentage of the above-mentioned standard value per specimen. Figure 1 summarises the study design, using one toothpaste as an example.

To facilitate categorising the abrasivity of the tested toothpastes, the classification suggested by Imfeld et al^15^ is adopted in this study. This classification subdivides RDA values into five groups as follows:

- RDA-1: Very low abrasion, RDA < 20
- RD-2: Low abrasion, RDA 20–40
- RDA-3: Moderate abrasion, RDA 40–60
- RDA-4: Strong abrasion, RDA 60–80
- RDA-5: Very strong abrasion, RDA > 80

Due to the fact that REA is derived from RDA and the standard abrasive is given the value 10, the following categories for REA could be arbitrarily suggested:

- REA-1: Very low abrasion, REA < 2
- REA-2: Low abrasion, REA 2–4
- REA-3: Moderate abrasion, REA 4–6
- REA-4: Strong abrasion, REA 6–8
- REA-5: Very strong abrasion, REA > 8

### Statistical Analysis

Each toothpaste was tested on a group of eight specimens. The mean value of each group represented the measured RDA or REA value of the respective toothpaste. A statistical comparison between the different toothpastes was not performed. The reason for this was the fact that the p-values would have had to be adjusted for multiple comparisons. Comparing 20 toothpastes would result in 190 comparisons giving a p-value of 0.0003. This might result in falsely statistically non-significant differences even for clinically large differences in RDA and REA values.

## Results

Table 1 shows the RDA and REA values measured in this study, RDA values declared by the manufacturers, RDA values measured by Tawakoli et al^17^ and abrasives utilised.

**Table 1:** RDA and REA values measured in this study, RDA values declared by the manufactures, RDA values measured by Tawakoli et al^17^ and the utilised abrasives

Toothpastes (manufacturer)	REA value 2019 (±SD)	RDA value 2019 (±SD)	Declared RDA	RDA value 20151 (±SD)	Abrasives*
ApaCare Remineralisierende Zahncreme (Cumdente; Tübingen, Germany)	7 ± 1	26 ± 2	50	-	SiO_2_ • *n*H_2_O Ca_5_(PO_4_)3OH
Candida Fresh Gel (Mibelle; Buchs, Switzerland)	3 ± 1	74 ± 8	50	75 ± 12	SiO_2_ • *n*H_2_O CaHPO_4_ • 2H_2_O
Candida Multicare 7 in 1 (Mibelle)	9 ± 2	81 ± 8	50	80 ± 3	SiO2 • *n*H_2_O
Candida Parodin (Mibelle)	2 ± 2	29 ± 2	25	24 ± 4	CaHPO_4_ • 2H_2_O Ca_5_(PO_4_)3OH SiO_2_ • *n*H_2_O
Candida Peppermint (Mibelle)	1 ± 1	42 ± 4	20	43 ± 3	CaHPO_4_ • 2H_2_O SiO_2_ • *n*H_2_O
Candida White Diamond (Mibelle)	244 ± 76	12 ± 2	30	-	SiO_2_ • *n*H_2_O Diamond powder
Candida White Micro-Crystals (Mibelle)	19 ± 3	84 ± 6	75	90 ± 10	SiO_2_ • *n*H_2_O
Colgate Dentagard Original (Colgate-Palmolive; New York, NY, USA)	3 ± 2	60 ± 6	-	78 ± 5	SiO_2_ • *n*H_2_O
Colgate Fresh Gel (Colgate-Palmolive)	4 ± 1	34 ± 3	-	33 ± 6	SiO_2_ • *n*H_2_O
Colgate Total Original (Colgate-Palmolive)	4 ± 2	100 ± 5	-	121 ± 7	SiO_2_ • *n*H_2_O
Dr. Wolff's Biorepair (Dr. Kurt Wolff; Bielefeld, Germany)	16 ± 6	131 ± 19	-	-	Zinc-Ca_5_(PO_4_)3OH SiO_2_ • *n*H_2_O SiO_2_
Elmex Kariesschutz (Colgate-Palmolive)	12 ± 1	69 ± 5	-	65 ± 3	SiO_2_ • *n*H_2_O
Elmex Sensitive Plus (Colgate-Palmolive)	7 ± 1	26 ± 3	-	28 ± 4	SiO_2_ • *n*H_2_O
Elmex Sensitive Professional (Colgate-Palmolive)	3 ± 2	29 ± 2	-	38 ± 3	CaCO_3_
Emoform F Diamond (Dr. Wild; Muttenz, Switzerland)	51 ± 25	42 ± 6	30	-	SiO_2_ Diamond powder
Meridol (Colgate-Palmolive)	11 ± 5	59 ± 8	-	65 ± 7	SiO_2_ • *n*H_2_O
Signal Anti-Caries (Unilever Dept ER; Leatherhead, UK)	3 ± 1	71 ± 6	50	108 ± 6	SiO_2_ • *n*H_2_O
Signal Micro-Granuli (Unilever Schweiz; Thayngen, Switzerland)	1 ± 1	43 ± 4	37	44 ± 4	SiO_2_ • *n*H_2_O
Signal White System (Unilever Schweiz)	8 ± 1	143 ± 6	110	110 ± 14	CaCO_3_ SiO_2_ • *n*H_2_O
Swiss Smile Diamond Glow (Curadent; Kriens, Switzerland)	177 ± 70	14 ± 1	20	-	SiO_2_ • *n*H_2_O Ca_5_(PO_4_)3OH Diamond powder SiO_2_

* Abrasives as declared by manufacturer: SiO_2_ • *n*H_2_O: hydrated silica; Ca_5_(PO_4_)3OH: hydroxyapatite; CaHPO_4_ • 2H_2_O: dicalcium phosphate dihydrate; SiO_2_: silica; CaCO_3_: calcium carbonate.

Signal White System (143 ± 6) and Dr. Wolff’s Biorepair (131 ± 19) scored the highest RDA values. Candida White Diamond (12 ± 2) and Swiss Smile Diamond Glow (14 ± 1) scored the lowest RDA values. The highest REA was observed for Candida White Diamond (244 ± 76) and Swiss Smile Diamond Glow (177 ± 70). Candida Peppermint (1 ± 1) and Signal Micro-Granuli (1 ± 1) scored the lowest REA.

Table 2 shows the ranking of the tested toothpastes based on their RDA and REA.

**Table 2:** Tested toothpastes ranked based on their RDA and REA values (RDA classified according to Imfeld et al^15^)

RDA-1: very low abrasive (RDA < 20)	REA-1: very low abrasive (REA < 2)
(1) Candida White Diamond (2) Swiss Smile Diamond Glow	(1) Candida Peppermint (2) Signal Micro-Granuli
**RDA-2: low abrasive (RDA 20-40)**	**REA-2: low abrasive (REA 2-4)**
(3) ApaCare Remineralisierende Zahncreme (4) Elmex Sensitive Plus (5) Candida Parodin (6) Elmex Sensitive Professional (7) Colgate Fresh Gel	(3) Candida Parodin (4) Candida Fresh Gel (5) Elmex Sensitive Professional (6) Signal Anti-Caries (7) Colgate Dentagard Original (8) Colgate Fresh Gel (9) Colgate Total Original
**RDA-3: moderate abrasive (RDA 40-60)**	**REA-3: moderate abrasive (REA 4-6)**
(8) Candida Peppermint (9) Emoform F Diamond (10) Signal Micro-Granuli (11) Meridol (12) Colgate Dentagard Original	
**RDA-4: strong abrasive (RDA 60-80)**	**REA-4: strong abrasive (REA 6-8)**
(13) Elmex Kariesschutz (14) Signal Anti-Caries (15) Candida Fresh Gel	(10) ApaCare Remineralisierende Zahncreme (11) Elmex Sensitive Plus (12) Signal White System
**RDA-5: very strong abrasive (RDA > 80)**	**REA-5: very strong abrasive (REA > 8)**
(16) Candida Multicare 7 in 1 (17) Candida White Micro-Crystals (18) Colgate Total Original (19) Dr. Wolff's Biorepair (20) Signal White System	(13) Candida Multicare 7 in 1 (14) Meridol (15) Elmex Kariesschutz (16) Dr. Wolff's Biorepair (17) Candida White Micro-Crystal (18) Emoform F Diamond (19) Swiss Smile Diamond Glow (20) Candida White Diamond

REA classification arbitrarily derived from RDA. Lower numbers indicate lower ranking.

**Fig 3:**
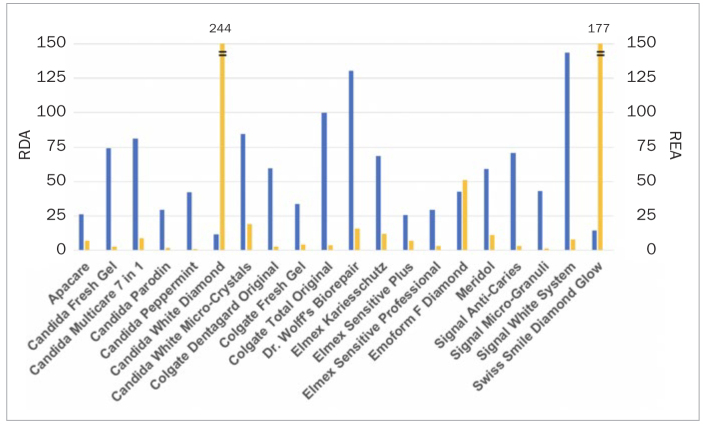
Combined presentation of mean RDA and REA values for each toothpaste (RDA: blue bars, REA: yellow bars). For clarity, the bars for REA were terminated at a value of 150 and standard deviation is not presented. The measured REA value for Candida White Diamond is 244 and for Swiss Smile Diamond Glow is 177.

It was noteworthy that the two toothpastes ranked 1st and 2nd based on their RDA were ranked 19th and 20th based on their REA (Candida White Diamond and Swiss Smile Diamond Glow). However, toothpastes with high RDA did not necessarily exhibit low REA values.

Interestingly, only three of the tested toothpastes have the same RDA as those determined by Tawakoli et al^17^ and those declared by the manufacturer: Candida Parodin (in 2015 marketed as Candida Parodin Professional; same formula [manufacturer’s informaion]), Candida White Micro-Crystals and Signal Micro-Granuli. Candida Fresh Gel, Candida Multicare 7 in 1 and Candida Peppermint had the same RDA in this study as in Tawakoli et al.^17^ However, both values are higher than the one declared by the manufacturer (see Table 1). The RDA values in 2015 (Tawakoli et al^17^) and the present study (2019) remained in the same range for the following toothpastes: Colgate Fresh Gel, Elmex Kariesschutz, Elmex Sensitive Plus and Meridol. Colgate Total Original, Colgate Dentagard Original and Signal Anti-Caries showed a 21%, 30% and 52% decrease, respectively, in RDA between 2015 and 2019. On the other hand, Signal White System and Elmex Sensitive Professional showed a 23% and 31% increase in their RDA value between 2015 and 2019, respectively.

## Discussion

Toothpastes play a major role in oral hygiene, but are also known to play a role in tooth wear. Studies have shown an increased prevalence and incidence of tooth wear which – among other things – calls for a better understanding of the mechanical properties of toothpastes on the market. This will help dentists and oral hygienists to advise their patients about the toothpaste most suited to their individual needs.^11^ Recently, toothpastes with diamond powder were introduced on the market claiming to exhibit low RDA. However, these and other toothpastes were not tested for their REA. The present study determined the RDA and REA of 20 toothpastes and tested whether toothpastes with diamond powder behave differently on dentin and enamel.

Permanent bovine mandibular incisors were used in this study. Bovine teeth have proven to be valid substitutes for human teeth in abrasion studies. They are larger, possess flatter surfaces and are easier to obtain than human teeth.^2,13,19^ No significant difference in the susceptibility to toothbrush abrasion was observed between human and bovine enamel^2^ or between human and bovine dentin.^19^

The first attempts to determine abrasivity of toothpastes on dentin used dentin weight loss as the index of abrasivity.^10,12^ Due to difficulties in controlling water loss from dentin specimens, weight-loss methods were discontinued.^3^ Measuring RDA and REA overcomes the water-loss issue, as it does not involve weighing as an index of abrasivity. The hardness of dentin and enamel remains unchanged after irradiation, and radioactive ^32^P is uniformly distributed throughout the specimen.^9^ Cerenkov radiation was measured in this study. This kind of radiation is subject to colour quenching. This means that the intrinsic colour of the radioactive slurry can resorb some of the released photons, thus lowering the measured radioactivity. To overcome this effect, a correction factor was calculated for each tested toothpaste. The correction factor is determined by measuring the radioactivity of a liquid containing the above-mentioned synthetic apatite, which was irradiated with the specimens simultaneously, and compares its radioactivity to the same liquid containing the same amount of synthetic apatite mixed with the respective toothpaste slurry. Although well standardised, RDA and REA methods shows certain variabilities. González-Cabezas et al^8^ mentioned that the RDA for the same toothpaste tested at different times could vary as much as 20%. This kind of variation has also been noticed by the experienced personnel in our laboratory. Furthermore, it should always be borne in mind that RDA and REA can vary considerably between different laboratories.^8^ It is therefore advisable that manufacturers state the laboratory in which RDA and/or REA were measured. This will help dentists to categorise toothpastes correctly.

To determine RDA and REA, sound dentin or enamel specimens are brushed, which does not always correspond to the clinical situation. Clinically, teeth are exposed to acid attacks that modify the hardness of enamel or dentin, and thus modify the way they are affected by abrasives in toothpastes. Wegehaupt et al^18^ found that enamel is less affected by diamond abrasives after an acid attack. The horizontal reciprocating brushing movement used in the RDA and REA method is in no way the one recommended to patients. Other brushing techniques (e.g. vertical or rotary) will have a modulating effect on toothpaste abrasivity.^15^ This also applies to different speeds and pressures used by patients while brushing, different toothbrush properties and the presence of dental pellicle and plaque. All of the above-mentioned factors emphasise the importance of being conservative when predicting the true clinical abrasivity of toothpastes based on their RDA or REA. However, RDA and REA are the only standardised parameters to express the abrasivity of toothpastes on dentin and enamel.

In this study, three toothpastes were categorised as highly abrasive on dentin, i.e. RDA > 80: Signal White System, Dr. Wolff`s Biorepair and Colgate Total Original. Toothpastes utilising diamond powder as abrasives, namely Candida White Diamond, Emoform F Diamond and Swiss Smile Diamond Glow, were categorised as only slightly or very slightly abrasive (RDA < 40). However, they exhibit by far the highest REA values (244, 177 and 51, respectively). The low RDA of toothpastes with diamond powder could be attributed to the fact that very hard diamond particles slip through the relative soft dentin rather than cutting it. A rather non-scientific but very comprehensible illustration is provided for the behavior of diamond particles on rubber (dentin) and glass (enamel). When rubbing diamond particles on a piece of rubber, the rubber will rather give way than become scratched. In other words, the diamond particles will rather sink into the soft rubber than cut it. On the other hand, when rubbing diamond particles on glass, glass is easily scratched.

Among the toothpastes with diamond powder, Emoform F Diamond showed the lowest REA (51 ± 25) and the highest RDA (42 ± 6). This REA value is still 2.5 times higher than the highest REA value of toothpastes with traditional abrasives (19 ± 3). The higher RDA of Emoform F Diamond compared to other toothpastes with diamond powder could be due to the different amounts of traditional abrasives in them.

The tested toothpastes containing diamond powder show extremely high REA values up to 244 ± 76 for Candida White Diamond, 177 ± 70 for Swiss Smile Diamond Glow and 51 ± 25 for Emoform F Diamond. To the best of our knowledge, no study has yet been carried out to determine the REA of any of the 20 toothpastes tested in this study. The high REA of toothpastes with diamond powder is not the only interesting finding in this study. Five other toothpastes show a very strong abrasivity on enamel (REA > 8). Three of these five toothpastes are also ranked as very strong abrasives on dentin (RDA > 80). Elmex Kariesschutz and Signal White System also show high RDA and high REA (see toothpastes ranked as strong and very strong abrasives for both RDA and REA in Table 2). The fact that a toothpaste exhibits high RDA and REA can raise the question of whether patients suffering from tooth wear should be dissuaded from using such a toothpaste. Concerning the ingredients of toothpastes exhibiting high RDA and REA, hydrated silica is found to be used as an abrasive in all of them. This should not lead to the false assumption that hydrated silica is automatically responsible for such high REA values.

Due to their inert nature, silica-based abrasives are widely used in toothpastes. They do not interact with fluoride ions.^5^ Ten of the toothpastes tested in this study contain silica-based particles as the only abrasive. However, the RDA and REA of these toothpastes vary considerably (RDA between 26 and 100, REA between 1 and 19). This variability could be attributed to the fact that the abrasivity of silica-based particles depends on many factors, such as particle amount, size and shape, water content and agglomeration degree.^5^ Another factor that could modify the abrasivity of silica particles in toothpastes are the surfactants (e.g. sodium lauryl sulphate). Surfactants alone have also been reported to cause some abrasion of dentin.^16^

Based on our increasing knowledge of RDA and REA and the fact that they shows a certain variability, a slight modification of the abrasivity classification presented by Imfeld et al^15^ could be suggested. The new suggestion is based on the idea that a 3-group classification – rather than 5-group – might be a more reliable and feasible approach for daily clinical practice for both dentists and patients. The new classification consists of the following groups:

- RDA/REA-1: Low abrasion, RDA < 40 (REA < 4)
- RDA/REA-2: Moderate abrasion, RDA 40–80 (REA 4–8)
- RDA/REA-3: High abrasion, RDA > 80 (REA > 8)

However, it should be noted that other schools of thought adopt different classifications of RDA values. An RDA classification in Germany labels toothpastes as highly abrasive only when their RDA exceeds 150. Moreover, listing toothpastes with an REA value of 9 in the same category as toothpastes with REA value of 177 and 244 – as in this study – seems to be unrealistic. A new concept for classifying toothpastes based on their REA and RDA might be advisable. A petition was lately raised to the European Parliament calling for a mandatory indication for the abrasivity of toothpastes. This shall be forwarded to working groups on cosmetic products and might initiate a further regulation in the future.^6^

The role of brushing technique has already been mentioned. Basically, modifying any factor in the toothbrushing equation will modify the resulted amount of tooth wear. To provide patients suffering from tooth wear with adequate tertiary prevention, a combination of suitable toothpaste, suitable toothbrush and a proper brushing technique should be prescribed. Knowing the RDA and REA of toothpastes is an important step towards prevention. However, further studies should be carried out to investigate suitable toothbrush-toothpaste combinations.

## Conclusion

This study confirms that toothpastes with diamond powder exhibit low RDA, while behaving extremely abrasively on enamel, i.e. they exhibit very high REA values. Patients should be informed accordingly.
